# Association between flavonoid and subclasses intake and metabolic associated fatty liver disease in U.S. adults: Results from National Health and Nutrition Examination Survey 2017–2018

**DOI:** 10.3389/fnut.2022.1074494

**Published:** 2022-12-01

**Authors:** Junlu Tong, Yingjuan Zeng, Jianhui Xie, Kecen Xiao, Man Li, Li Cong

**Affiliations:** ^1^Department of Endocrinology and Metabolism, The Fifth Affiliated Hospital of Sun Yat-sen University, Zhuhai, Guangdong, China; ^2^Guangdong Provincial Key Laboratory of Biomedical Imaging and Guangdong Provincial Engineering Research Center of Molecular Imaging, The Fifth Affiliated Hospital, Sun Yat-sen University, Zhuhai, Guangdong, China; ^3^Center for Interventional Medicine, The Fifth Affiliated Hospital, Sun Yat-sen University, Zhuhai, Guangdong, China

**Keywords:** flavonoid, metabolic associated fatty liver disease, anthocyanins, isoflavones, NHANES

## Abstract

**Background:**

Metabolic associated fatty liver disease (MAFLD) formerly known as non-alcoholic fatty liver disease (NAFLD) is the most common chronic liver disease. Flavonoid is considered a promising candidate for metabolic disease prevention although few studies have explored the relationship between flavonoid intake and MAFLD.

**Purpose:**

To assess the relationship between flavonoid intake and MAFLD prevalence in the U.S. adult population.

**Materials and methods:**

The data of this cross-sectional study was obtained from National Health and Nutrition Examination Survey (NHANES) and Food and Nutrient Database for Dietary Studies (FNDDS) 2017–2018. Flavonoid and subclasses intake was assessed by two 24h recalls. MAFLD was diagnosed according to the consensus definitions. Multivariate logistic regression model was performed to examine the association between flavonoid intake and MAFLD with adjustments for confounders.

**Results:**

A total of 4,431 participants were included in this cross-sectional analysis. MAFLD had a weighted prevalence of 41.93% and was not associated with total flavonoid intake. A higher anthocyanin and isoflavone intake, on the other hand, was associated with a lower prevalence of MAFLD. The protective effect of higher anthocyanin intake was significant among male, Non-Hispanic White, and Non-Hispanic Asia participants. Higher isoflavone intake was associated with a lower risk of MAFLD in participants of younger (age < 50), Non-Hispanic Black, Non-Hispanic Asia, and higher HEI-2015 scores compared with the lowest quartile of isoflavone intake. Stratified analysis showed that compared with the lowest quartile of anthocyanin intake, the effect of anthocyanin intake on MAFLD varied by racial groups (*P*_*interaction*_ = 0.02). A positive correlation existed between HDL and anthocyanidin intake (*P* = 0.03), whereas a negative correlation existed between FPG and isoflavone intake (*P* = 0.02).

**Conclusion:**

MAFLD was adversely linked with flavonoid subclasses, anthocyanin and isoflavone. This modifiable lifestyle provides a potential opportunity to prevent MAFLD. These findings promote future research into the links and mechanisms between anthocyanin and isoflavone intake and MAFLD.

## Introduction

With an estimated prevalence of 25 to 45%, non-alcoholic fatty liver disease (NAFLD) is one of the most common chronic liver diseases globally, putting an enormous burden on the health and economic systems (1, 2). Metabolic Associated Fatty Liver Disease (MAFLD) is considered to be a more appropriate concept than NAFLD given the growing prevalence of metabolic dysfunction (3). Patients with MAFLD were diagnosed with a definite criterion instead of exclusion conditions, which is distinguished from NAFLD. Several recent studies have pointed out that MAFLD has a higher prevalence and a more severe liver injury compared with NAFLD (4, 5). There is no doubt that MAFLD is a major health problem worldwide and will likely become a serious public health issue if the government does not act. There is currently no clinically approved medication with proven effectiveness for MAFLD. Lifestyle modifications such as increasing exercise and changing diet may be useful in the early stage of MAFLD (6). Therefore, investigating the potential dietary strategies for MAFLD could contribute to the prevention and progression of this disease.

The pathogenesis of NAFLD results in a series of ‘multiple hits’ hypothesis (7). Although the mechanism of NAFLD is complex, containing abdominal adiposity, insulin resistance, inflammation, and other factors. Notably, oxidative stress, which generates excessive reactive oxygen species (ROS), is involved in the entire development of NAFLD (8). Flavonoids are natural compounds found in many fruits, vegetables, red wine, and tea. There are several subclasses of flavonoids, such as anthocyanins, isoflavones, flavones, flavanones, flavan-3-ols, and isoflavones. Due to the effect of attenuating oxidative stress and inflammation, flavonoids present an attractive therapy for hepatic steatosis (9). Previous studies showed that the prevalence of metabolic-related diseases was lower with higher flavonoid intake (10–12).

Several studies of *in vitro* and animal model experiments confirmed the potential effect of flavonoids in the prevention and progression of NAFLD (13). Ying et al. found that high-fat diet-induced rats treated with flavonoids could alleviate insulin resistance and inflammation, and significantly enhance the levels of antioxidants (14). A clinical trial reported that patients who received supplementation of anthocyanin, a subclass of flavonoid, could markedly decrease plasma levels of ALT and glucose in NAFLD individuals (15). The NHANES 2005-2010 result showed an inverse relationship between flavonoid intake and the risk of NAFLD (16). Moreover, a prospective study of the Chinese population pointed out that higher flavonoid intake reduced the risk of NAFLD progression in elderly obese or overweight individuals (17). These results suggest that flavonoids could be beneficial to NAFLD patients. Nevertheless, previous NAFLD studies could not accurately depict the association between flavonoid intake and MAFLD because MAFLD is more heterogeneous than NAFLD. To our knowledge, there was no clinical evidence linking flavonoids and subclasses intake to MAFLD. Thus, we aim to investigate the association between flavonoids and subclasses intake with MAFLD prevalence in this cross-section study.

## Materials and methods

### Study design and participants

**FIGURE 1:**
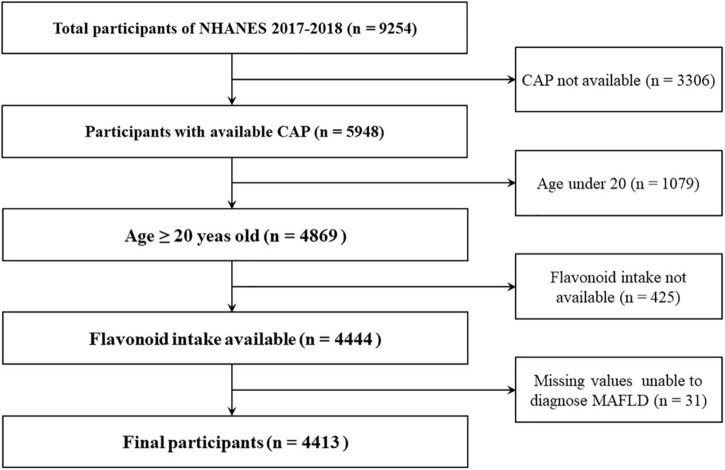
Flow chart of participants selection. NHANES, National Health and Nutrition Examination Survey; CAP, controlled attenuation parameter.

The data of all participants was obtained from the database of the National Health and Nutrition Examination Survey (NHANES) and the United States Department of Food and Nutrient Database for Dietary Studies (FNDDS) during the cycle of 2017-2018. There are 9,254 participants in the cycle of NHANES. We excluded the participants for the following reasons: missing the median values of controlled attenuation parameter (CAP), age < 20 years old, missing flavonoid data, and insufficient information to diagnose MAFLD. Finally, a total of 4,413 participants were included in our study and the flowchart was shown in Figure 1.

The NHANES research, which has been approved by the National Center for Health Statistics and Research Ethics Review Committee, is a cross-sectional survey designed to measure the health and nutrition of the U.S. adult population. Informed consent forms have been completed by all participants.

### Study variables and outcome

Based on the clinical experience and recent literature (5), variables that may be related to flavonoid intake and MAFLD were collected and analyzed in this study. The variables included age, sex, race, educational level, poverty to income ratio (PIR), smoking status, alcohol drinking status (heavy, moderate, mild, and never); body mass index (BMI); diabetes (no, borderline or yes); hypertension (no or yes); and hyperlipidemia (no or yes). Laboratory markers, including fasting plasma glucose (FPG, mmol/L), alanine aminotransferase (ALT, IU/L), aspartate aminotransferase (AST, IU/L), triglyceride (TG, mmol/L), high-density lipoprotein (HDL, mg/dl), and hypersensitive C-reactive protein (Hs-CRP, mg/L) were quantified with NHANES methodology standards.

The dietary intake information was collected from participants for up to two 24-h recall interviews during NHANES. The first dietary recall was performed at the visit interview and the second was accomplished by telephone interview 3-10 days later. The values of flavonoid and subclasses intake were calculated using the United States Department of Food and Nutrient Database for Dietary Studies (FNDDS), which is linked to NHANES and using the same method to estimate the energy intake (kcal/day) and healthy eating index (HEI)-2015 score. These dietary data were calculated by the average of the two 24h-recall interviews (one day was used if only one was available). In order to evaluate the attribution of flavonoid intake in the comprehensive diet categories, we use the covariate of HEI-2015 scores which is a calculated value of dietary quality for individual dietary intake to analyze the effect of flavonoid intake on MAFLD prevalence (18).

Outcome status is based upon the consensus concerning definitions of MAFLD (19). Clinicians frequently employ vibration-controlled transient elastography (VCTE) as a quick and accurate test. During the 2017-2018 period, participants were examined with VCTE using FibroScan^®^ model 502 V2 Touch equipped devices by NHANES staff. The CAP values of 274 dB/m, which separated S0 from S1-3 with 90% sensitivity, could detect the existence of hepatic steatosis (20). Hepatic steatosis was found to be present, and one of the three following criteria: overweight or obesity, signs of metabolic dysregulation, and the presence of type 2 diabetes mellitus (T2DM) was also present to determine the diagnosis of MAFLD (19). Overweight or obesity was defined by BMI ≥ 25kg/m^2^ in Caucasians or BMI ≥ 23kg/m^2^ in Asians. There was metabolic dysregulation when at least two of the following conditions were met: (1) waist circumference ≥ 102/88 cm in men/women (or ≥ 90/80 cm in Asian men/women); (2) blood pressure ≥ 130/85 mmHg or treatment with a specific drug; (3) plasma level of TG ≥ 150 mg/dl or treatment with a specific drug; (4) plasma level of HDL < 40 mg/dl in men or < 50 mg/dl in women or treatment with a specific drug; (5) prediabetes (FPG 5.6- 6.9 mmol/L, or 2-h post-load glucose levels 7.8-11.0 mmol/L or HbA1c 5.7-6.4%); (6) homeostasis model assessment of insulin resistance score ≥ 2.5; and (7) plasma level of Hs-CRP > 2 mg/L (21). T2DM is defined as the participants who had DM as reported by a doctor or in accordance with the American Diabetes Association guidelines (22). The diagnosis of hypertension and hyperlipidemia included the patients with a history informed by professionals and the participants found in the examination according to the diagnostic criteria (23, 24).

### Statistical analysis

The appropriate NHANES sample weight was applied to the complex surgery design. The data were described as *n* (%) for categorical variables, mean (standard deviation, SD) for continuous variables with a normal distribution, and median with interquartile spacing (IQR, Q1-Q3) for continuous variables with non-normal distribution. Continuous variables were assessed for normality using the Kolmogorov–Smirnov test. The differences of continuous variables between two groups were identified by Student’s *t*-test and Mann-Whitney Wilcoxon Test for normal and non-normal distribution. The flavonoid, anthocyanidin and isoflavone intake were analyzed by quartiles for non-normal distribution and Kruskal Wallis test was used to evaluate the difference among quartile groups for continuous variables. The chi-square was used to evaluate the intergroup difference for the categorical variables. The association between flavonoid, anthocyanidin or isoflavone intake and the risk of MAFLD was investigated using three models of weighted multivariate logistic regression analyses. Model 1 was adjusted for no covariates, model 2 was adjusted for age, sex, and race. In model 3, we additionally adjusted for education level, PIR, smoking status, alcohol drinking status, total energy, and HEI-2015 scores. The weighted smooth curve fitting was performed after adjustment for covariates to display the relationship between anthocyanidin and isoflavone intake and the risk of MAFLD. The stratified analysis was classified by age, sex, race, and HEI-2015 scores (the cut off was the median of HEI-2015 scores). The stratified analysis further explored the heterogeneity of the effect of anthocyanidins and isoflavones on the risk of MAFLD. Then, the interaction model was constructed to study whether the interaction between anthocyanidins or isoflavones and other variables existed. Finally, the correlation between indexes of liver injury and metabolism and anthocyanidin and isoflavone intake was analyzed using generalized linear regression. All statistical tests were two-sided and completed using R (Version 4.2) and EmpowerStats Software^1^. *P* < 0.05 was considered statistically significant.

## Results

### Participants baseline characteristics

A total of 4,413 participants with 1945 (41.93%) were classified as MAFLD according to the criteria. Compared to the non-MAFLD, MAFLD participants were often men, elder, Mexican-American, and more likely to be accompanied with overweight/obesity (BMI ≥ 25kg/m^2^), diabetes, hypertension, and hyperlipidemia. Besides, they had the characteristic of enhanced BMI, higher plasma levels of FPG, ALT, AST, TG, Hs-CRP, and lower plasma level of HDL. They also took more energy per day and had unhealthy diet habits (lower HEI-2015 scores). Although the median levels of total flavonoid and subclasses intake were lower among MAFLD compared to non-MAFLD participants, only anthocyanidin (*P* = 0.001) and isoflavone (*P* = 0.01) intake had a statistically significant difference between MAFDL and non-MAFLD individuals (Table 1).

**TABLE 1:** Characteristics of participants by the metabolic associated fatty liver disease (MAFLD) status, weighted.

Characteristic	Non-MAFLD	MAFLD	*P*-value
	(*n* = 2,468)	(*n* = 1,945)	
**Demographic**			
Age (years), mean (SD)	45.69 (0.72)	51.38 (0.73)	<0.0001
**Sex, *n* (%)**			<0.0001
Female	1,352 (55.26)	872 (44.18)	
Male	1,116 (44.74)	1,073 (55.82)	
**Race, *n* (%)**			<0.0001
Non-Hispanic White	852 (64.70)	701 (62.79)	
Non-Hispanic Black	676 (12.65)	372 (8.96)	
Non-Hispanic Asian	350 (5.38)	235 (4.98)	
American Mexican	235 (5.87)	356 (12.14)	
Other/Multi-Racial	355 (11.40)	281 (11.13)	
**Education level, *n* (%)**			0.11
Less than high school	446 (10.24)	376 (10.38)	
High school	575 (25.83)	493 (30.32)	
College and high	1,447 (63.94)	1,076 (59.30)	
PIR, poverty income ratio, mean (SD)	3.11 (0.06)	3.09 (0.07)	0.79
**Smoking status, *n* (%)**			0.01
Never	1,445 (58.47)	1,074 (55.59)	
Former	521 (23.16)	561 (29.08)	
Current	502 (18.37)	310 (15.32)	
**Taking alcohol status, *n* (%)**			0.17
Never	795 (24.42)	642 (26.48)	
Mild	834 (36.66)	693 (38.28)	
Moderate	432 (19.91)	271 (15.27)	
Heavy	407 (19.01)	339 (19.97)	
**BMI level, *n* (%)**			<0.0001
<25 kg/m2	1,017 (42.84)	105 (3.45)	
≥ 25 kg/m2	1,451 (57.16)	1,840 (96.55)	
**Diabetes, *n* (%)**			<0.0001
No	2,027 (87.80)	1,134 (62.51)	
Boundary	133 (5.40)	179 (10.52)	
Yes	308 (6.80)	632 (26.97)	
**Hypertension, *n* (%)**			<0.0001
No	1,523 (71.76)	821 (44.48)	
Yes	945 (28.24)	1,124 (55.52)	
**Hyperlipidemia, *n* (%)**			<0.0001
No	1,023 (43.88)	370 (20.10)	
Yes	1,444 (56.12)	1,575 (79.90)	
**Examination results, mean (SD)**			
BMI (kg/m^2^)	26.71 (0.23)	34.06 (0.40)	<0.0001
FPG (mmol/L)	5.16 (0.02)	6.03 (0.06)	<0.0001
ALT (IU/L)	20.00 (0.49)	27.67 (0.69)	<0.0001
AST (IU/L)	21.59 (0.44)	23.30 (0.46)	0.02
TG (mmol/L)	1.30 (0.02)	2.07 (0.06)	<0.0001
HDL (mg/dl)	57.47 (0.51)	47.96 (0.60)	<0.0001
Hs-CRP (mg/L)	2.99 (0.19)	4.97 (0.26)	<0.0001
**Dietary measures, mean (SD)**			
Total energy (kcal/day)	2,063.86 (22.10)	2,164.22 (25.86)	0.02
HEI-2015 scores	52.92 (0.92)	50.55 (0.65)	0.01
**Flavonoid and subclasses intake (mg/day), median (IQR)**			
Total flavonoids	71.41 (24.63, 245.09)	64.87 (22.55, 199.63)	0.22
Anthocyanidins	2.57 (0.05, 16.78)	1.21 (0.00, 8.70)	0.001
Flavan-3-ols	18.22 (4.65, 176.96)	17.51 (4.55, 143.23)	0.56
Flavanones	0.32 (0.01, 7.64)	0.22 (0.00, 7.35)	0.05
Flavones	0.50 (0.15, 1.19)	0.46 (0.12, 1.09)	0.17
Flavonols	13.94 (6.99, 24.42)	12.84 (6.86, 24.35)	0.74
Isoflavones	0.02 (0.00, 0.18)	0.01 (0.00, 0.07)	0.01

SD, standard deviation; IQR, Interquartile range; BMI, body mass index; FPG, Fasting plasma glucose; ALT, Alanine aminotransferase; AST, Aspartate aminotransferase; TG, triglyceride; HDL, high-density lipoprotein; Hs-CRP, hypersensitive C-reactive protein; HEI, healthy eating index.

### The quartile analysis of flavonoid and subclasses intake associated with covariates

To test the relationship between flavonoids and MAFLD, we analyzed the total of flavonoid intake by quartiles. However, the prevalence of MAFLD is not significantly different among quartile groups of total flavonoid intake (*P* = 0.28). A higher level of flavonoid intake was positively correlated with plasma level of HDL, and negatively correlated with BMI and plasma level of ALT, TG, Hs-CRP (Supplementary Table S1). The quartile analysis of anthocyanidin and isoflavone intake showed that variables of race, education, RIP, BMI, smoking status, alcohol drinking status, BMI, HEI-2015 scores, energy intake and plasma level of Hs-CRP were significantly different in both anthocyanidins and isoflavones quartile groups. Moreover, the variables of age, sex, plasma levels of ALT, TG, HDL were different among anthocyanidin intake quartiles, and plasma level of FPG had a statistically significant difference among isoflavone intake quartiles (Table 2).

**TABLE 2:** The weighted association between anthocyanidin and isoflavone intake and other covariates.

	Anthocyanidins					Isoflavones				
Variable	Quartile 1	Quartile 2	Quartile 3	Quartile 4	*P*-value	Quartile 1	Quartile 2	Quartile 3	Quartile 4	*P*-value
Median (Range) (mg/day)	0.00 (0.00-0.02)	0.40 (0.02-1.59)	3.93 (1.59-11.16)	33.85 (11.16-643.83)		0.00 (0.00-0.00]	0.00 (0.00-0.01)	0.03 (0.01-0.11)	1.59 [0.11-390.60]	
**Demographic**										
Age group, *n* (%)					<0.001					0.16
<50 years	583 (61.25)	506 (54.02)	458 (47.98)	454 (46.55)		760 (53.75)	245 (47.09)	443 (49.98)	553 (55.35)	
≥ 50 years	545 (38.75)	584 (45.98)	634 (52.02)	649 (53.45)		896 (46.25)	350 (52.91)	620 (50.02)	546 (44.65)	
**Sex, *n* (%)**					0.002					0.64
Female	494 (43.80)	554 (50.19)	561 (49.80)	615 (58.04)		836 (50.55)	314 (53.64)	513 (49.30)	561 (50.21)	
Male	634 (56.20)	536 (49.81)	531 (50.20)	488 (41.96)		820 (49.45)	281 (46.36)	550 (50.70)	538 (49.79)	
**Race, *n* (%)**					0.02					<0.001
Non-Hispanic Asian	112 (4.09)	131 (5.14)	150 (5.34)	192 (6.19)		157 (4.01)	41 (2.32)	155 (6.03)	232 (7.77)	
Non-Hispanic Black	317 (13.73)	272 (11.69)	238 (10.75)	221 (8.47)		452 (13.46)	181 (13.25)	219 (9.04)	196 (8.43)	
American Mexican	108 (6.66)	158 (9.68)	201 (11.13)	124 (6.76)		193 (7.54)	60 (7.21)	180 (10.11)	158 (9.12)	
Other/Multi-Racial	145 (9.98)	145 (9.27)	176 (13.44)	170 (12.32)		228 (10.50)	79 (9.56)	169 (12.35)	160 (12.40)	
Non-Hispanic White	446 (65.54)	384 (64.22)	327 (59.34)	396 (66.27)		626 (64.49)	234 (67.66)	340 (62.48)	353 (62.28)	
**Education level, *n* (%)**					<0.0001					0.002
Less than high school	241 (12.80)	218 (10.53)	232 (12.20)	131 (6.03)		347 (12.21)	77 (7.18)	224 (11.92)	174 (7.86)	
High school	334 (34.52)	287 (32.70)	248 (26.38)	199 (18.18)		460 (32.44)	169 (29.34)	226 (23.41)	213 (23.97)	
College and high	553 (52.68)	585 (56.76)	612 (61.42)	773 (75.79)		849 (55.35)	349 (63.48)	613 (64.67)	712 (68.17)	
RIP, mean (SD)	2.80 (0.10)	2.95 (0.08)	3.07 (0.08)	3.54 (0.06)	<0.0001	2.86 (0.09)	3.23 (0.10)	3.15 (0.09)	3.32 (0.07)	<0.001
**Smoking status, *n* (%)**					<0.0001					0.03
Never	547 (48.05)	623 (56.87)	648 (60.69)	701 (63.04)		866 (53.26)	340 (57.55)	618 (56.92)	695 (63.07)	
Former	251 (23.51)	259 (25.03)	294 (27.04)	278 (26.89)		419 (25.63)	137 (23.54)	278 (29.90)	248 (23.01)	
Now	330 (28.44)	208 (18.10)	150 (12.27)	124 (10.07)		371 (21.11)	118 (18.91)	167 (13.18)	156 (13.92)	
**Alcohol drinking status, *n* (%)**					<0.001					0.04
Never	389 (26.22)	361 (25.91)	386 (31.89)	301 (17.83)		580 (27.69)	188 (22.16)	324 (22.35)	345 (26.24)	
Mild	334 (33.34)	361 (33.97)	375 (37.49)	457 (43.89)		515 (34.38)	184 (32.63)	424 (44.43)	404 (37.76)	
Moderate	179 (17.24)	173 (18.85)	158 (13.85)	193 (21.61)		267 (17.97)	110 (22.24)	144 (14.58)	182 (18.65)	
Heavy	226 (23.20)	195 (21.27)	173 (16.77)	152 (16.66)		294 (19.97)	113 (22.97)	171 (18.64)	168 (17.35)	
**BMI group, *n* (%)**					0.02					<0.001
<25 kg/m2	281 (23.79)	247 (23.40)	273 (25.94)	321 (31.62)		390 (23.82)	142 (23.42)	262 (24.13)	328 (33.44)	
≥ 25 kg/m2	847 (76.21)	843 (76.60)	819 (74.06)	782 (68.38)		1266 (76.18)	453 (76.58)	801 (75.87)	771 (66.56)	
**Diabetes, *n* (%)**					0.29					0.14
No	811 (75.93)	749 (73.72)	775 (78.51)	826 (80.26)		1184 (75.14)	416 (74.09)	733 (77.15)	828 (81.86)	
Borderline	88 (8.00)	83 (9.31)	77 (6.75)	64 (6.29)		110 (8.34)	53 (10.34)	78 (6.34)	71 (5.97)	
Yes	229 (16.07)	258 (16.97)	240 (14.74)	213 (13.45)		362 (16.52)	126 (15.57)	252 (16.51)	200 (12.17)	
**Hypertension, *n* (%)**					0.18					0.05
No	595 (59.01)	571 (58.53)	577 (58.76)	601 (64.54)		840 (57.71)	300 (57.00)	561 (62.43)	643 (63.94)	
Yes	533 (40.99)	519 (41.47)	515 (41.24)	502 (35.46)		816 (42.29)	295 (43.00)	502 (37.57)	456 (36.06)	
**Hyperlipidemia, *n* (%)**					0.34					0.21
No	369 (33.91)	329 (30.52)	347 (33.59)	348 (37.20)		486 (32.48)	200 (31.75)	326 (33.96)	381 (37.08)	
Yes	759 (66.09)	761 (69.48)	744 (66.41)	755 (62.80)		1170 (67.52)	394 (68.25)	737 (66.04)	718 (62.92)	
MAFLD, *n* (%)					0.004					0.01
No	615 (51.67)	590 (57.06)	593 (57.19)	670 (65.70)		904 (56.35)	321 (50.33)	574 (58.72)	669 (64.19)	
Yes	513 (48.33)	500 (42.94)	499 (42.81)	433 (34.30)		752 (43.65)	274 (49.67)	489 (41.28)	430 (35.81)	
**Examination results, mean (SD)**										
FPG (mmol/L)	5.53 (0.05)	5.54 (0.05)	5.50 (0.08)	5.51 (0.09)	0.98	5.61 (0.06)	5.75 (0.13)	5.49 (0.07)	5.30 (0.05)	0.004
ALT (IU/L)	24.39 (0.81)	24.11 (0.91)	23.53 (0.93)	21.06 (0.65)	0.04	23.58 (0.98)	24.28 (1.42)	23.31 (0.91)	22.03 (0.37)	0.16
AST (IU/L)	22.43 (0.49)	22.69 (0.59)	22.96 (0.63)	21.25 (0.49)	0.16	22.84 (0.66)	22.48 (0.81)	21.73 (0.52)	21.97 (0.49)	0.19
TG (mmol/L)	1.66 (0.07)	1.73 (0.07)	1.65 (0.04)	1.47 (0.06)	0.002	1.61 (0.04)	1.72 (0.08)	1.73 (0.06)	1.49 (0.04)	0.06
HDL (mg/dl)	50.59 (0.64)	52.43 (0.61)	53.36 (0.86)	57.20 (0.86)	<0.0001	53.34 (0.74)	53.17 (1.22)	52.27 (0.66)	54.94 (0.77)	0.32
Hs-CRP (mg/L)	4.49 (0.33)	4.32 (0.45)	3.48 (0.16)	3.08 (0.21)	0.01	4.36 (0.31)	4.05 (0.27)	3.43 (0.18)	3.28 (0.24)	0.004
BMI (kg/m^2^)	31.11 (0.42)	30.14 (0.30)	29.79 (0.43)	28.28 (0.30)	<0.0001	30.48 (0.31)	30.27 (0.33)	29.35 (0.35)	28.95 (0.54)	0.003
**Dietary measures, mean (SD)**										
HEI-2015 scores	42.05 (0.67)	49.35 (0.55)	54.85 (0.57)	60.71 (0.63)	<0.0001	48.38 (0.85)	52.30 (0.96)	54.99 (0.82)	53.98 (0.82)	<0.0001
Energy (Kcal/day)	2,001.60 (30.09)	2,059.85 (33.59)	2210.27 (28.41)	2148.51 (42.36)	0.001	1,987.34 (21.81)	2,019.39 (42.77)	2,225.46 (36.33)	2214.40 (48.59)	<0.0001

SD, standard deviation; BMI, body mass index; FPG, Fasting plasma glucose; ALT, Alanine aminotransferase; AST, Aspartate aminotransferase; TG, triglyceride; HDL, high-density lipoprotein; Hs-CRP, hypersensitive C-reactive protein; HEI, healthy eating index.

### Higher anthocyanidin and isoflavone intake was associated with lower risk of metabolic associated fatty liver disease

**FIGURE 2:**
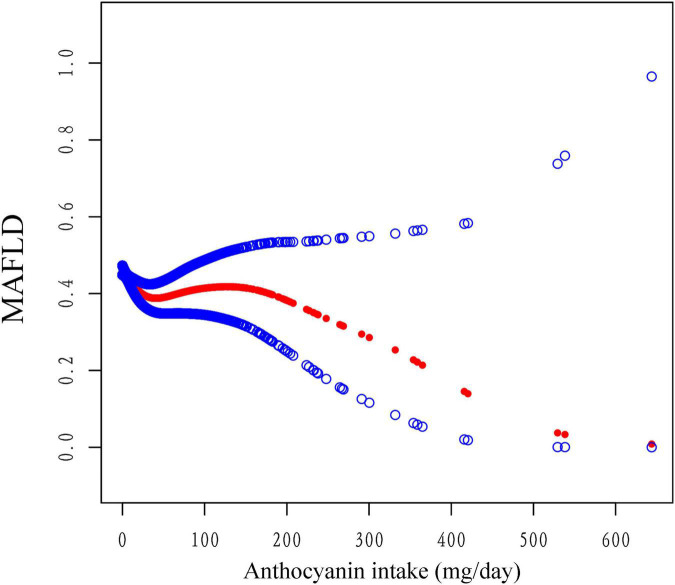
The association between anthocyanidin intake and the risk of MAFLD using smooth curve fitting, weighted. The covariates of age, sex, race, PIR, smoking status, alcohol drinking status, education level, total energy and HEI-2015 scores were adjusted. The red points line represented the fitting spline. The blue points line represented the 95% confidence intervals.

**FIGURE 3:**
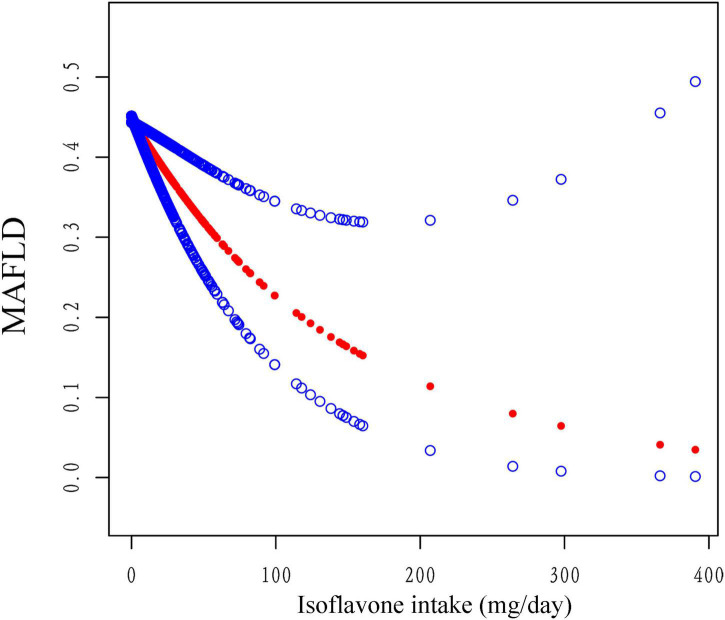
The association between isoflavone intake and the risk of MAFLD using smooth curve fitting, weighted. Age, sex, race, PIR, smoking status, alcohol drinking status, education level, total energy and HEI-2015 scores were adjusted. The red points line represented the fitting spline. The blue points line represented the 95% confidence intervals.

Multiple logistic regression models indicated that the third quartile (Q3) and the fourth quantile (Q4) of anthocyanidin intake had a lower risk of MAFLD than the first quantile in model 2 [Odds Radio (OR), OR_*Q*3_ = 0.66, 95% confidence interval (CI): 0.46-0.94; OR_*Q*4_ = 0.47, 95% CI: 0.35-0.63, *P*_*Trend*_ < 0.001] and model 3 (OR_*Q*3_ = 0.70, 95%CI: 0.53-0.93; OR_*Q*4_ = 0.56, 95%CI: 0.42-0.74, *P*_*Trend*_ < 0.001), while there were no statistically significant differences between the second quantile (Q2) (Table 3). The analysis showed that Q4 of isoflavone intake reduced the risk of MAFLD comparing to Q1 in model 1 (OR = 0.55, 95%CI: 0.41-0.74). After adjustment of covariates, there was no significant difference between different quartiles of isoflavone intake. Moreover, in model 3, Q2 of isoflavone intake even increased the risk of MAFLD comparing to Q1 (OR = 1.36, 95%CI: 1.02-1.81) (Table 3). We performed the smooth curve fitting further revealed the negative relationship between anthocyanidin and isoflavone intake and the risk of MAFLD (Figures 2, 3). To further verify the robustness of the results, participants with missing data (*n* = 813) were removed from the analysis. Multivariable logistic regression analysis further demonstrated higher anthocyanidin intake decreased the risk of MAFLD (Supplementary Table S2). Moreover, BMI was one of the diagnostic criteria in MAFLD and the indexes of metabolic dysregulation was associated with diabetes, hypertension and hyperlipidemia, we additionally analyzed the correlation of anthocyanidin and isoflavone intake with the risk of MAFLD in participants combined with these metabolic disorders (Supplementary Table S3). The results showed that higher anthocyanidin intake decreased the risk of MAFLD in participants combined with overweight/obesity (BMI ≥ 25kg/m^2^) and hyperlipidemia (*P* < 0.05). The Q4 of isoflavone intake is associated with lower MAFLD prevalence in diabetes mellitus comparing to Q1 (OR = 0.54, 95%CI: 0.29-1.00).

**TABLE 3:** The multivariate logistic regression analysis results of association between anthocyanidin or isoflavone intake with the risk of metabolic associated fatty liver disease (MAFLD) prevalence, weighted.

Variable	Quartile 1	Quartile 2	Quartile 3	Quartile 4	*P-*trend
**Anthocyanidins**					
Median (Range) (mg/day)	0.00 [0.00-0.02]	0.40 (0.02-1.59)	3.93 (1.59-11.16)	33.85 (11.16-643.83)	
Model1 [OR (95% CI)]	Referent	0.80 (0.58-1.12)	0.80 (0.59-1.08)	**0.55 (0.41-0.74)**	0.001
Model2 [OR (95% CI)]	Referent	0.72 (0.49-1.06)	**0.66 (0.46-0.94)**	**0.47 (0.35-0.63)**	<0.001
Model3 [OR (95% CI)]	Referent	0.74 (0.52-1.05)	**0.70 (0.53-0.93)**	**0.56 (0.42-0.74)**	<0.001
**Isoflavones**					
Median (Range) (mg/day)	0.00 (0.00-0.00]	0.00 (0.00-0.01]	0.03 (0.01-0.11)	1.59 [0.11-390.60]	
Model1 [OR (95% CI)]	Referent	1.27 (0.96-1.69)	0.91 (0.69-1.19)	**0.72 (0.54-0.97)**	0.02
Model2 [OR (95% CI)]	Referent	1.28 (0.91-1.81)	0.85 (0.62-1.18)	0.71 (0.51-1.00)	0.02
Model3 [OR (95% CI)]	Referent	1.36 (1.02-1.81)	0.92 (0.70-1.20)	0.76 (0.57-1.02)	0.03

OR, odds ratio; 95% CI, 95% confidence interval. Model 1: No covariates were adjusted. Model 2: Age, sex, and race were adjusted. Model 3: Age, sex, race, PIR, smoking status, alcohol drinking status, education level, total energy and HEI 2015 scores were adjusted. The bold values refer to *P* < 0.05 indicated significant statistical difference.

**FIGURE 4:**
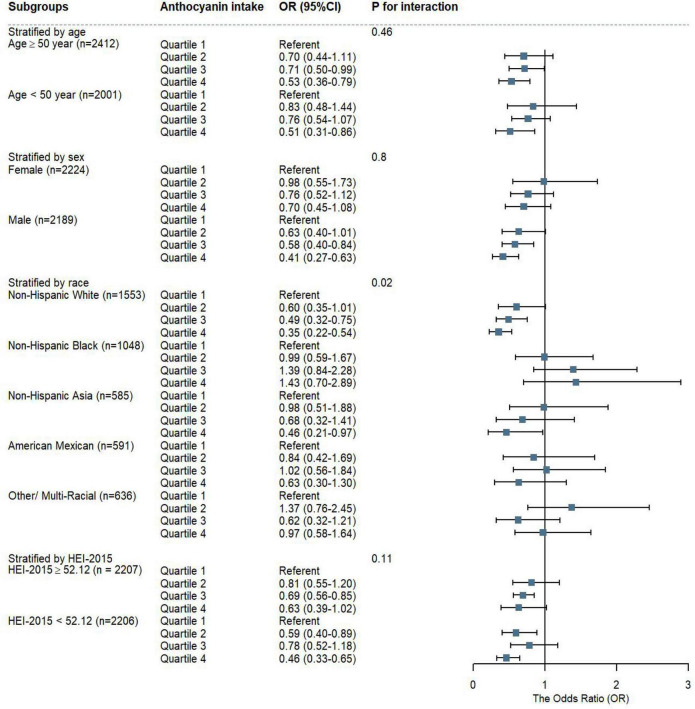
The weighted stratified and interaction analysis of association between anthocyanidin intake and covariates. The results were adjusted for covariates of age group, sex, race, educational level, PIR, smoking status, alcohol drinking status, energy intake, HEI-2015 scores, except for the corresponding variables.

We discovered that, when compared to Q1, Q4 of anthocyanidin intake was significant associated with a lower risk of MAFLD in participants of male (OR = 0.41, 95%CI: 0.27-0.63), Non-Hispanic White (OR = 0.35, 95%CI: 0.22-0.54), and Non-Hispanic Asia (OR = 0.46, 95%CI: 0.21-0.97). The negative correlation between anthocyanidin intake and MAFLD prevalence was robust in participants of different age groups and HEI-2015 levels. The interaction results showed that the effect of anthocyanidin intake on MAFLD prevalence varied by racial groups (*P* = 0.02) (Figure 4). The risk of MAFLD was reduced in Q4 of isoflavone intake comparing to Q1 in participants of younger (age < 50 years old) (OR = 0.54, 95%CI: 0.37-0.79), Non-Hispanic Black (OR = 0.74, 95%CI: 0.55-0.99), Non-Hispanic Asia (OR = 0.43, 95%CI: 0.26-0.71), and higher scores of HEI-2015 (OR = 0.57, 95%CI: 0.38-0.86). There was no interaction effect of these covariates with isoflavone intake on MAFLD prevalence (Supplementary Figure 1).

### Anthocyanidin and isoflavone intake was associated with markers of liver injury and metabolism

The results showed there was a significant positive correlation between HDL and anthocyanidin intake and the standard β coefficient was 0.100 (*P* < 0.05). The isoflavone intake was negatively associated with the plasma level of ALT, FPG and positively linked with the plasma level of AST standard, the β coefficient was −0.0470, −0.2919, and 0.0509, respectively (Table 4).

**TABLE 4:** Adjusted linear regression of liver and metabolism markers and anthocyanidin or isoflavone intake, weighted.

	Anthocyanidins		Isoflavones	
Variables	Standard β	*P*-value	Standard β	*P*-value
AST (U/L)	–0.0338	0.62	0.0509	0.03
ALT (U/L)	–0.0971	0.07	–0.0470	0.01
FPG (mmol/L)	–0.4790	0.18	–0.2919	0.02
HDL (mg/dl)	0.1000	0.03	0.0139	0.38
TG (mmol/L)	0.0007	1.00	–0.0810	0.65
Hs-CRP (mg/L)	–0.1072	0.18	0.0023	0.91

The results were adjusted for covariates of age, sex, race, educational level, PIR, smoking status, alcohol drinking status, energy intake, HEI-2015 scores. FPG, Fasting plasma glucose; ALT, Alanine aminotransferase; AST, Aspartate aminotransferase; TG, triglyceride; HDL, high-density lipoprotein; Hs-CRP, hypersensitive C-reactive protein; HEI, healthy eating index.

## Discussion

The overall weighted prevalence of MAFLD was 41.93%, suggesting that the soaring epidemic of this disease has caused a serious burden on public health. Hence, the primary prevention of MAFLD through dietary and behavioral modification is an urgent need. This is the first study reporting the association between flavonoid and subclasses intake and the risk of MAFLD in U.S. adults based on NHANES. Although the higher total flavonoid intake was not associated with a lower prevalence of MAFLD, the anthocyanidin intake had an inverse association with the risk of MAFLD. Moreover, the protective effect of higher anthocyanidin intake was more significant in male, Non-Hispanic White, Non-Hispanic Asia, overweight or obesity, and individuals combined with hyperlipidemia. Higher isoflavone intake may have a benefit to prevent MAFLD in individuals of younger, Non-Hispanic Black, Non-Hispanic Asia and higher HEI-2015 scores. The racial groups had an interaction effect with anthocyanin intake on the risk of MAFLD. There was a positive correlation between HDL and anthocyanidin intake, and a negative correlation between FPG and isoflavone intake. These results promise that dietary modification of increasing anthocyanidin and isoflavone intake provides a possible prevention approach for MAFLD, especially in particular groups.

The pathogenic driver of MAFLD is characterized by the liver’s inability to handle the energy substrates leading to toxic lipid accumulation (25). The dysfunction in lipid metabolism leads to the activation of oxidative stress, stimulation of ROS, the release of pro-inflammatory cytokines and apoptotic cell death, and subsequent stimulation of inflammation and fibrogenesis (26). Based on previous evidence from *in vitro* and *in vivo* experiments, flavonoids could be beneficial in inhibiting the occurrence and progression of NAFLD, and the mechanism is mainly related to their anti-inflammatory and antioxidant effect (16, 17). In our study, a higher level of flavonoid intake was benefit to improve the MAFLD-related indicators, such as HDL, ALT, TG, and Hs-CRP, however, there was no difference in total flavonoid intake between non-MAFLD and MAFLD individuals. The findings implied that total flavonoid intake is not an independent risk factor for MAFLD and that various subclasses of components may have different effects. More emphasis should be placed on the association between flavonoid subclasses and MAFLD.

According to the FNDDS database, there are six subclasses of flavonoids as follows have been evaluated: anthocyanidins, flavanones, flavones, flavan-3-ols, flavanols, and isoflavones. Our study showed that two subclasses of flavonoids, anthocyanidins and isoflavones, could be potential protective factors in the risk of MAFLD, and this difference was significant in anthocyanidins. This reason might be the higher consumed of anthocyanin intake in the daily diet of the U.S. population (27). The most common source of anthocyanidins are berries, appealing vegetables, and cereals. Previous studies reported that anthocyanidin treatment could increase insulin secretion and decrease the level of plasma LDL and hepatic TG contents as antioxidants in obese mice or rats (28, 29). Blueberry polyphenols improved obesity and metabolic alterations in high-fat diet induced mice through modulation of the gut microbiota (30). Further studies illustrated that cherry anthocyanidins are protected against NAFLD via activating the autophagy pathway (31). In summary, anthocyanins could prevent and improve NAFLD, which has been confirmed *in vitro* and animal experiments. However, few clinical studies reported the effects of anthocyanidins on NAFLD/MAFLD. A randomized double-blind clinical study showed that a supplement of anthocyanin decreased liver injury biomarkers and improved insulin resistance and clinical evolution of NAFLD patients (32). Our results show that anthocyanin intake is significantly higher in patients with non-fatty liver and negatively correlated with the risk of MAFLD. This result is an important supplement to the correlation between anthocyanins and MAFLD.

We discovered that the higher anthocyanidin intake with a lower risk of MAFLD was obvious in men, Non-Hispanic White, Non-Hispanic Asia, overweight/obesity and hyperlipidemia participants. A large number of studies have proved that man, age, and obesity are independent risk factors associated with hepatic steatosis (33–35). In animal and human studies, young and female individuals are protected in dysmetabolism resulting from the ability to convert excess fatty acids into ketone body production and enhanced sex-specific browning (36). It’s possible that estrogen deficiency exacerbated hepatic steatosis and inflammatory changes in mice and zebrafish models (37, 38). Obesity was closely associated with the prevalence and severity of NAFLD and advanced disease. When the capacity of adipose tissue to store energy was overloaded, hepatocytes store the extra lipids, leading to ectopic fat accumulation, insulin resistance, and hepatic steatosis (39). The result of the generalized linear regression model analysis was in line with the result of multiple regression analysis. Race as an independent risk factor for MAFLD enhanced the effect of anthocyanin intake on MAFLD in our results. The positive correlation between HDL and anthocyanidin intake could explain that higher anthocyanidin intake is protective against MAFLD in participants combined with hyperlipemia. Although dietary intervention alone was unlikely to prevent the incidence and progression of MAFLD, the findings that anthocyanins had a protective role against MAFLD, especially in these subgroups, provides a practical preventive measure to minimize the risk of MAFLD. Moreover, many studies have confirmed that dietary polyphenol is a new target to prevent NAFLD. Daily intake of 1,500 mg curcumin could reduce serum cholesterol, glucose, and ALT in patients with NAFLD (40). A study of systematic review and meta-analysis showed that curcumin, naringenin, hesperidin, catechin, and silymarin may have therapeutic efficacy in patients with NAFLD (41). The result of our study on anthocyanins is complementary to the research on the correlation between polyphenols and MAFLD.

Isoflavones have been shown in animal models and *in vitro* studies to protect against NAFLD via multiple mechanisms that modulate oxidative stress, lipid synthesis, fatty acid β-oxidation, peroxisome proliferator-activated receptor α (PPARα) activity, and aldose reductase/polyol production (42). Dietary soy isoflavones could prevent the accumulation of hepatic lipid droplets and delay the progression of NAFLD due to the regulation of lipogenesis and lipolysis, suppression of hepatic PPARγ2, and promotion of fatty acid oxidation (43, 44). There were limited epidemiological studies reporting the association of isoflavone intake with NAFLD. A Chinese cohort epidemiological study showed that higher soy food intake ≥ 4 times/week reduced the 25% prevalence of NAFLD in Chinese adults (45). Another study using the data from Chinese adults in the Nutrition Health Atlas Project reported that total isoflavones intake was inversely associated with NAFLD, hyperlipidemia, and hypertension (46). The protective effect of higher isoflavones intake in Asia individuals for MAFLD prevalence was in line with the Chinese cohort studies. Several studies have found that higher diet quality (higher scores of HEI-2015) was associated with a lower risk of NAFLD (47, 48). Our study discovered that higher isoflavone intake was associated with a lower risk of MAFLD among participants with higher HEI-2015 scores. This finding supported prior research and suggested that higher isoflavone intake might benefit from a higher diet quality. Previous studies have shown that young and Black individuals have lower risk of NAFLD (49). We found that higher isoflavones intake was negatively associated with MALFD prevalence in these subgroups, indicating that isoflavones might be a marginally effective intervention in MAFLD prevention. The mechanism of higher isoflavone intake was protective against MAFLD in the context of diabetes since isoflavones activated PPAR, which played a central role in the regulation of blood glucose homeostasis and insulin sensitivity (50). This could be the reason for negative relationship between isoflavone intake and plasma level of FPG. Besides, there was a negative association between isoflavone intake with plasma level of ALT but a positive association with AST, both ALT and AST are the markers of liver injury, so isoflavone intake could not beneficial in liver injury. Moreover, we found that the Q2 of isoflavone intake increased the risk of MAFLD. A previous study in line with our result, the supplement of daidzein in Zucker fatty rats did not decrease the body weight and serum leptin, liver steatosis scores, and energy intake did not show significant differences either (51). Another study investigated the effect of 2-heptyl-formononetin (C7F) and formononetin, an O-methylated isoflavone, on lipid metabolism in C57BL/6J mice. The diet-supplemented formononetin or C7F increased the level of triglycerides and induced hepatic steatosis compared with a cholesterol-enriched diet (52). However, a randomized double-blind controlled trial showed that patients of NAFLD daily supplemented with 250 mg genistein for 8-weeks could improve fat metabolism and reduce insulin resistance (53). As a result, the effect of isoflavone intake on fatty liver disease was controversial. More research is required to establish the isoflavone impact on MAFLD and the underlying mechanism.

To our knowledge, this is the first national population-based study to evaluate the relationship between flavonoid and subclasses (especially anthocyanins and isoflavones) intake and the risk of MAFLD. With the large-scale representative survey data, our analysis results are effective and reliable for adjusting the potential confounding variables. The relationship between anthocyanin or isoflavone intake and the risk of MAFLD was evaluated in diverse individual groups. This may give a diet suggestion for the prevention of MAFLD and metabolic diseases. However, the study’s limitations should be acknowledged. Firstly, this study is a cross-sectional investigation that could only identify relationships rather than causality. Secondly, a person’s long-term diet intake was not well represented by the 24-h recall, and MAFLD may have occurred before they provided the interview data, implying that the bias of flavonoid intake was unavoidable. Thirdly, our results only represent the U.S. population. Although this survey included results from multiple racial groups, flavonoid intake may vary substantially depending on the diet habits and dietary components of different regions. Furthermore, despite multiple confounding variables have been adjusted, residual confounding unknown to us may affect the results. Finally, further research is needed to corroborate our findings and the underlying mechanisms.

## Conclusion

In summary, this study provided evidence that higher anthocyanin intake is associated with a lower risk of MAFLD for the U.S. adult population. There was a negative relationship between isoflavone intake and MAFLD prevalence in participants of younger (age < 50), Non-Hispanic Black, Non-Hispanic Asia, and higher scores of HEI-2015. These results imply that higher anthocyanin and isoflavone intake could prevent MAFLD in the dietary modification. Further studies are warranted to elucidate our findings and confirm the mechanism.

## Data availability statement

The original contributions presented in this study are included in the article/Supplementary material, further inquiries can be directed to the corresponding authors.

## Ethics statement

The studies involving human participants were reviewed and approved by Institutional Review Board of the National Center for Health Statistics (NCHS). The patients/participants provided their written informed consent to participate in this study.

## Author contributions

JT designed the research, collected the data, performed the statistical analysis, and drafted the manuscript. YZ investigated and curated data. JX and KX contributed to the conception and literature search. LC and ML supervised the statistical analysis, reviewed and edited the manuscript. All authors have approved the final manuscript.

## Footnotes

1
http://www.empowerstats.com
