# Supplementary material for: Association between flavonoid and subclasses intake and metabolic associated fatty liver disease in U.S. adults: Results from National Health and Nutrition Examination Survey 2017–2018
Source: Front Nutr. 2022 Dec 1;9:1074494. doi: 10.3389/fnut.2022.1074494 (PMC9751205; doi:10.3389/fnut.2022.1074494)
Supplement: Supplementary file 1 [file Data_Sheet_1.docx]

Supplementary Material

# Supplementary Tables

## Supplementary Tables S1

**Supplementary Table S1** The weighted association between the total flavonoid intake and other covariates.

| **Total flavonoid intake** | | | | | |
| --- | --- | --- | --- | --- | --- |
| **Variables** | **Quartile 1** | **Quartile 2** | **Quartile 3** | **Quartile 4** | ***P*-value** |
| Median (Range) (mg/day) | 11.43 [0.00- 22.25] | 39.04 (22.25- 62.46] | 104.57 (62.46-193.21] | 421.31 (193.21-5476.76] |  |
| **Demographic** |  |  |  |  |  |
| Age Group, n (%) |  |  |  |  | 0.02 |
| <50 years | 553 (57.80) | 507 (55.07) | 466 (46.65) | 475 (50.35) |  |
| ≥50 years | 551 (42.20) | 596 (44.93) | 637 (53.35) | 628 (49.65) |  |
| Sex, n (%) |  |  |  |  | 0.32 |
| Female | 535 (47.64) | 571 (53.41) | 556 (50.78) | 562 (50.61) |  |
| Male | 569 (52.36) | 532 (46.59) | 547 (49.22) | 541 (49.39) |  |
| Race, n (%) |  |  |  |  | < 0.0001 |
| Non-Hispanic Asian | 66 (2.56) | 120 (4.31) | 146 (5.53) | 253 (8.00) |  |
| Non-Hispanic Black | 309 (13.88) | 279 (12.72) | 255 (10.90) | 205 (7.47) |  |
| American Mexican | 161 (9.74) | 163 (9.63) | 169 (9.49) | 98 (5.55) |  |
| Other/ Multi-Racial | 163 (10.66) | 174 (12.31) | 184 (13.81) | 115 (8.67) |  |
| Non-Hispanic White | 405 (63.16) | 367 (61.03) | 349 (60.27) | 432 (70.31) |  |
| Education level, n (%) |  |  |  |  | < 0.0001 |
| Less than high school | 251 (13.19) | 219 (10.89) | 208 (9.86) | 144 (7.67) |  |
| High school | 334 (36.31) | 270 (27.01) | 254 (24.41) | 210 (23.84) |  |
| College and high | 519 (50.50) | 614 (62.10) | 641 (65.73) | 749 (68.49) |  |
| RIP, mean (SD) | 2.80 (0.10) | 2.95 (0.08) | 3.07 (0.08) | 3.54 (0.06) | < 0.0001 |
| Smoking status, n (%) |  |  |  |  | <0.0001 |
| never | 542 (45.64) | 626 (60.33) | 666 (60.84) | 685 (61.44) |  |
| former | 261 (27.98) | 282 (23.50) | 288 (27.53) | 251 (23.77) |  |
| now | 301 (26.38) | 195 (16.16) | 149 (11.62) | 167 (14.79) |  |
| Alcohol drinking status, n (%) |  |  |  |  | 0.01 |
| never | 378 (26.83) | 338 (24.02) | 365 (24.85) | 356 (25.42) |  |
| mild | 316 (31.31) | 377 (34.59) | 389 (38.70) | 445 (43.70) |  |
| moderate | 183 (18.13) | 181 (18.84) | 173 (18.77) | 166 (16.35) |  |
| heavy | 227 (23.72) | 207 (22.56) | 176 (17.67) | 136 (14.52) |  |
| BMI group, n (%) |  |  |  |  | 0.22 |
| <25 kg/m^2^ | 258 (22.34) | 271 (26.61) | 288 (27.65) | 305 (28.34) |  |
| ≥25 kg/m^2^ | 846 (77.66) | 832 (73.39) | 815 (72.35) | 798 (71.66) |  |
| Diabetes, n (%) |  |  |  |  | 0.97 |
| No | 772 (76.29) | 790 (76.64) | 807 (77.60) | 792 (78.10) |  |
| Borderline | 92 (7.90) | 67 (7.38) | 73 (7.83) | 80 (7.13) |  |
| Yes | 240 (15.81) | 246 (15.98) | 223 (14.57) | 231 (14.77) |  |
| Hypertension, n (%) |  |  |  |  | 0.82 |
| No | 587 (59.19) | 575 (59.36) | 594 (61.61) | 588 (60.95) |  |
| Yes | 517 (40.81) | 528 (40.64) | 509 (38.39) | 515 (39.05) |  |
| Hyperlipidemia, n (%) |  |  |  |  | 0.84 |
| No | 371 (34.20) | 338 (33.62) | 333 (32.74) | 351 (34.96) |  |
| Yes | 733 (65.80) | 765 (66.38) | 769 (67.26) | 752 (65.04) |  |
| MAFLD, n (%) |  |  |  |  | 0.28 |
| No | 602 (56.70) | 610 (56.34) | 623 (57.11) | 633 (61.62) |  |
| Yes | 502 (43.30) | 493 (43.66) | 480 (42.89) | 470 (38.38) |  |
| **Examination results**, mean (SD) | |  |  |  |  |
| FPG (mmol/L) | 5.53 (0.05) | 5.54 (0.05) | 5.50 (0.08) | 5.51 (0.09) | 0.98 |
| ALT (IU/L) | 24.39 (0.81) | 24.11 (0.91) | 23.53 (0.93) | 21.06 (0.65) | 0.04 |
| AST (IU/L) | 22.43 (0.49) | 22.69 (0.59) | 22.96 (0.63) | 21.25 (0.49) | 0.16 |
| TG (mmol/L) | 1.66 (0.07) | 1.73 (0.07) | 1.65 (0.04) | 1.47 (0.06) | 0.002 |
| HDL (mg/dl) | 50.59 (0.64) | 52.43 (0.61) | 53.36 (0.86) | 57.20 (0.86) | < 0.0001 |
| Hs-CRP (mg/L) | 4.49 (0.33) | 4.32 (0.45) | 3.48 (0.16) | 3.08 (0.21) | 0.01 |
| BMI (kg/m^2^) | 31.11 (0.42) | 30.14 (0.30) | 29.79 (0.43) | 28.28 (0.30) | < 0.0001 |
| **Dietary measures**, mean (SD) | |  |  |  |  |
| HEI-2015 scores | 42.05 (0.67) | 49.35 (0.55) | 54.85 (0.57) | 60.71 (0.63) | < 0.0001 |
| Energy (Kcal/day) | 2001.60 (30.09) | 2059.85 (33.59) | 2210.27 (28.41) | 2148.51 (42.36) | 0.001 |

SD, standard deviation; BMI, body mass index; FPG, Fasting plasma glucose; ALT, Alanine aminotransferase; AST, Aspartate aminotransferase; TG, triglyceride; HDL, high-density lipoprotein; Hs-CRP, hypersensitive C-reactive protein; HEI, healthy eating index.

## Supplementary Tables S2

**Supplementary Table S2** The multivariate logistic regression analysis results of association between anthocyanidin or isoflavone intake and the risk of MAFLD using data with no missing value, weighted (n=3600).

| **Variables** | **Quartile 1** | **Quartile 2** | **Quartile 3** | **Quartile 4** | ***P-*Trend** | |
| --- | --- | --- | --- | --- | --- | --- |
| **Anthocyanidins** |  |  |  |  |  | |
| Median (Range) (mg/day) | 0.00 [0.00-0.02] | 0.40 (0.02-1.62] | 3.93 (1.62-12.35] | 33.85 (12.35-643.83] |  | |
| Model1 [OR (95% CI)] | Referent | 0.83 (0.58-1.18) | 0.84 (0.63-1.13) | **0.58 (0.41-0.82)** | 0.005 |  |
| Model2 [OR (95% CI)] | Referent | 0.73 (0.48-1.12) | **0.68 (0.48-0.96)** | **0.48 (0.34-0.69)** | 0.001 |  |
| Model3 [OR (95% CI)] | Referent | 0.74 (0.50-1.10) | **0.71 (0.55-0.92)** | **0.53 (0.37-0.76)** | 0.002 |  |
| **Isoflavones** |  |  |  |  |  |  |
| Median (Range) (mg/day) | 0.00 (0.00-0.00] | 0.00 (0.00-0.01] | 0.03 (0.01-0.12) | 1.59 [0.12-390.60] |  |  |
| Model1 [OR (95% CI)] | Referent | 1.34 (0.95-1.90) | 0.92 (0.67-1.26) | 0.72 (0.52-1.00) | 0.03 |  |
| Model2 [OR (95% CI)] | Referent | 1.36 (0.90-2.06) | 0.86 (0.59-1.25) | 0.72 (0.49-1.06) | 0.04 |  |
| Model3 [OR (95% CI)] | Referent | 1.46 (1.03-2.07) | 0.93 (0.67-1.29) | 0.76 (0.54-1.08) | 0.06 |  |

OR, odds ratio; 95% CI, 95% confidence interval. Model 1: No covariates were adjusted. Model 2: Age, sex, and race were adjusted. Model 3: Age, gender, race, PIR, smoking status, alcohol drinking status, education level, total energy and HEI-2015 scores were adjusted. The bolds represented *P* < 0.05.

## Supplementary Tables S3

**Supplementary Table S3** The multivariate logistic regression analysis results of association between anthocyanidin or isoflavone intake and the risk of MAFLD in participants combined MAFLD-related metabolic disease, weighted.

| **Variables** | **Quartile 1** | **Quartile 2** | **Quartile 3** | **Quartile 4** |
| --- | --- | --- | --- | --- |
| **Anthocyanidins** |  |  |  |  |
| Overweight/obesity (BMI≥25 kg/m^2^) (n=3291) | Referent | 0.69 (0.50-0.96) | 0.77 (0.57-1.05) | **0.54 (0.40-0.73)** |
| Diabetes (n=940) | Referent | 0.71 (0.43-1.18) | 0.84 (0.56-1.26) | 0.98 (0.57-1.70) |
| Hypertension (n=2069) | Referent | 0.67 (0.42-1.08) | 1.01 (0.57-1.79) | 0.91 (0.54-1.54) |
| Hyperlipidemia (n=1393) | Referent | **0.67 (0.46-0.98)** | **0.69 (0.49-0.96)** | **0.52 (0.37-0.74)** |
| **Isoflavones** |  |  |  |  |
| Overweight/obesity (BMI≥25 kg/m2) (n=3291) | Referent | 1.37 (0.92-2.05) | 0.91 (0.67-1.25) | 0.85 (0.58-1.26) |
| Diabetes (n=940) | Referent | 1.02 (0.55-1.87) | 0.89 (0.47-1.71) | 0.54 (0.29-1.00) |
| Hypertension (n=2069) | Referent | **1.70 (1.05-2.75)** | 1.05 (0.72-1.54) | 1.05 (0.72-1.54) |
| Hyperlipidemia (n=1393) | Referent | 1.30 (0.92-1.83) | 0.94 (0.62-1.43) | 0.81 (0.59-1.12) |

The results were adjusted for covariates of age, sex, race, educational level, PIR, smoking status, alcohol drinking status, energy intake, HEI-2015 scores. The results were presented as OR (95%CI). BMI, body mass index; OR, odds ratio; 95% CI, 95% confidence interval. The bolds represented *P* < 0.05.

# Supplementary Figures


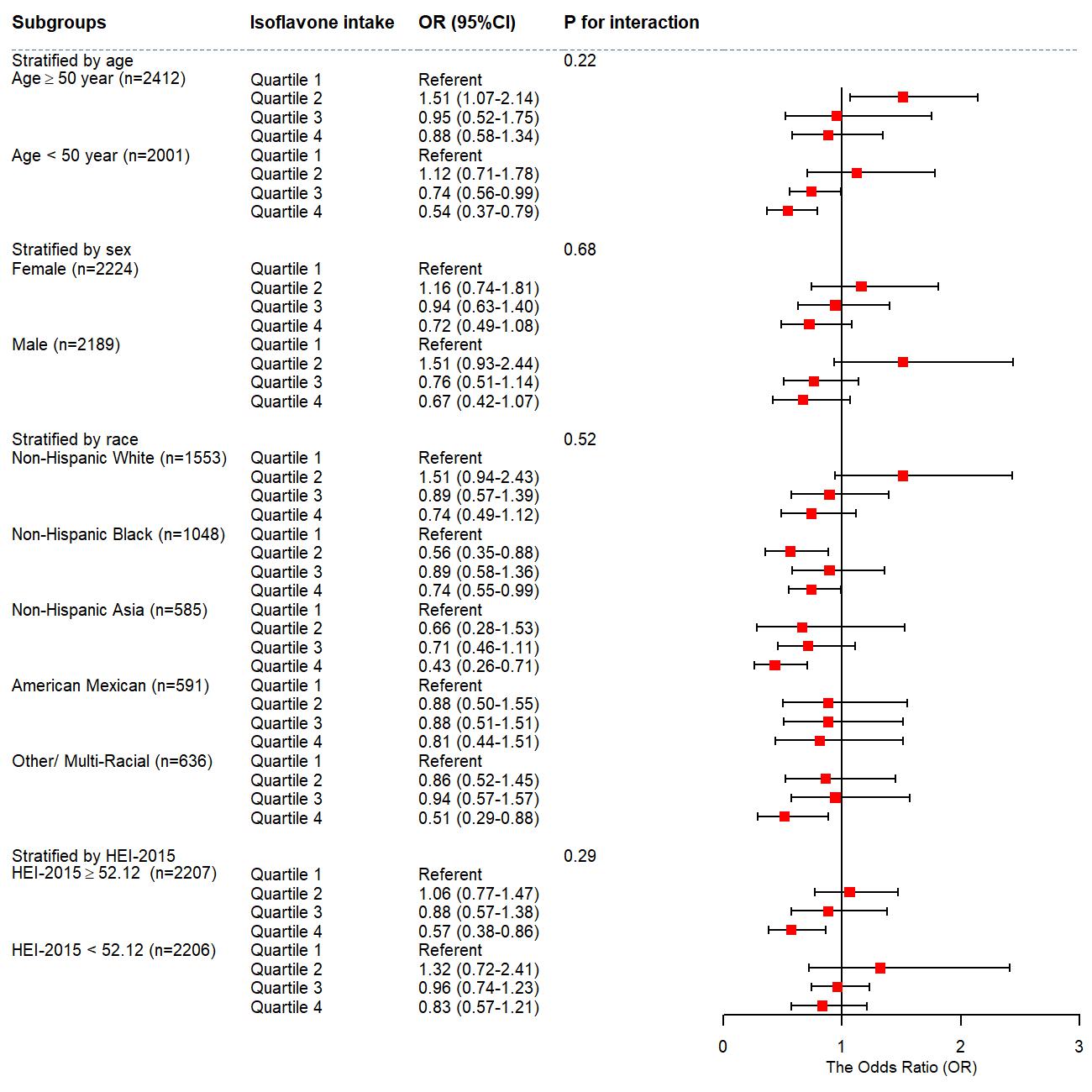


**Supplementary Figure 1**

The weighted stratified and interaction analysis of association between isoflavone intake and covariates. The results were adjusted for covariates of age group, sex, race, educational level, PIR, smoking status, alcohol drinking status, energy intake, HEI-2015 scores, except for the corresponding variables. OR, odds ratio; 95% CI, 95% confidence interval; HEI, healthy eating index.
